# Symptoms Contributing to Mobility Limitations and Fear of Falls in Older Adults.

**DOI:** 10.1093/geroni/igab046.1731

**Published:** 2021-12-17

**Authors:** Michelle McKay, Janell Mensinger, Melissa O'Connor, Alexander Costello, Suzanne Leveille

**Affiliations:** 1 Villanova University, Villanova University, Pennsylvania, United States; 2 Villanova University, Fitzpatrick College of Nursing, Villanova University, Pennsylvania, United States; 3 Villanova University, M. Louise Fitzpatrick College of Nursing, Villanova, Pennsylvania, United States; 4 University of Massachusetts Boston, University of Massachusetts Boston, Massachusetts, United States

## Abstract

Mobility limitations in older adults are associated with negative outcomes including fear of falls (FOF) and poorer quality of life. However, self-reported symptoms contributing to mobility difficulty have not been fully explored as an area for intervention. The study aimed to identify the prevalence of self-reported symptom causes of difficulty walking and stair-climbing. In addition, we examined associations between symptoms and FOF in a population-based cohort of community-dwelling older adults in the MOBILIZE Boston Study. Of the 243 older adults who reported difficulty with walking one quarter of a mile or climbing stairs, 67% were women, 72% were white, average age=79.4y (SD=5.7). FOF was measured with the Tinetti Falls Efficacy Scale. Pain was most commonly reported as the primary symptom responsible for mobility difficulty (38.4%) followed by endurance (21.1%), multiple symptoms (15.6%), weakness (13.2%), balance (8.7%), other symptoms (2.9%). Factorial ANCOVA determined gender differences in associations between symptoms and FOF, adjusting for age. In pairwise comparisons, women who identified balance as their primary symptom had higher FOF than women identifying endurance (p=.017), pain (p=.015), other (p=.017), or multiple (p=.050) symptoms. There were no FOF differences for women identifying balance compared to weakness as the primary issue (p=.395). Men who identified balance as their primary symptom had higher FOF than those who identified pain (p=.036); no other FOF differences were noted in men identifying balance compared to other symptoms. Understanding common symptoms experienced by older adults, and symptoms associated with greatest FOF, will assist in developing tailored interventions for mobility improvement.

